# Complicated *Trichosporon asahii* mastoiditis in immunocompetent child

**DOI:** 10.1186/s12879-021-06915-w

**Published:** 2021-12-07

**Authors:** Miral Al Momani, Dawood H. Yusef, Du’a Hamasha, Moh’d Rawhi Abu Hamad, Sara Farran

**Affiliations:** 1 Department of Pediatrics and Neonatology, Faculty of Medicine, Jordan University of Science and Technology, Irbid, Jordan; 2 Faculty of Medicine, Jordan University of Science and Technology, Irbid, Jordan

**Keywords:** *Trichosporon asahii*, Mastoiditis, Drug resistance, Children, Fungal, Case report

## Abstract

**Background:**

*Trichosporon asahii* is an opportunistic fungus that causes infections in immunosuppressed patients. It is rarely seen in children and immunocompetent hosts. The mortality rates are still high despite early treatment with proper antifungal drugs. *Trichosporon asahii* mastoiditis in an immunocompetent child makes this case challenging.

**Case presentation:**

This report presents a case of *Trichosporon asahii* mastoiditis which was complicated by transverse sinus thrombosis, in an otherwise healthy 21-month-old girl, and successfully treated with voriconazole. *Trichosporon asahii* was isolated, in three different occasions, from ear discharge of an immunocompetent healthy child, who presented with prolonged history of fever and received appropriate dosages of multiple types of antimicrobials as an outpatient but without improvement. After 48 h of starting the Voriconzole; post auricular swelling and ear discharge improved significantly.

**Conclusion:**

A high index of clinical and microbiological suspicion is needed for optimal diagnosis of *Trichosporon* infection. *Trichosporon asahii* can also cause infection in immunocompetent individual even without previous history of hospitalization or intervention. We emphasize the importance of early pediatric infectious evaluation and intervention**.**

## Background

*Trichosporon asahii* is non-candida yeast and an opportunistic organism. It is mainly affecting individuals with underlying hematologic malignancies and causes disseminated infection [1–4]. Although many cases of disseminated trichosporonosis have been reported in immunocompromised patients, few have been reported in persons without an underlying disease [5].

*Trichosporon* spp. is implicated in superficial and mucosal infections. However, systemic infections are seen in patients with predisposing factors for infection such as immunodeficiency, underlying malignancy, severe burns, transplant patients as well as patients on corticosteroid treatment, peritoneal dialysis, prolonged mechanical ventilation and those undergoing prosthetic valve surgeries [1, 2, 4, 6]. The mortality rates are still high despite early treatment with proper antifungal drugs [4].

We present the successful treatment of Trichosporon asahii mastoiditis in an otherwise healthy 21-month-old girl with Voriconasol.

## Case presentation

A 21-month-old girl presented to the emergency department as a case of left-sided mastoiditis with a history of prolonged, persistent fever, left side ear discharge and post auricular swelling. The patient was previously healthy; she had no previous admissions or surgeries and had uneventful antenatal period, she was delivered full term via normal vaginal delivery, and no neonatal intensive care unit (NICU) admission. She did not take any medications, was up to date with her vaccinations, and had no known allergies. She was doing well until she experienced fever for 25 days prior to admission. Her parent sought medical advice after 4 days of fever and she was treated as a case of tonsillitis with oral Amoxicillin and Clavulanate for 5 days. There was no improvement. In addition to fever, she developed runny nose, cough, and difficulty in breathing that were associated with decreased appetite and hypoactivity. The medication was switched to Azithromycin for 3 days. Despite the changes in antimicrobial drugs, the temperature remained high. Another private doctor prescribed Amoxicillin for another 3 days and the fever subsided just for 2 days at the time of starting Amoxicillin. After that, she developed pain in her left ear and bi-temporal headache as well. She received three doses of intramuscular Penicillin injection. But she did not show any improvement. Then she was diagnosed to have otitis media by Ear Nose Throat doctor and was given high dose of Amoxicillin and Clavulanate for 10 days but she continued to have fever and left ear pain.

Two days before her presentation to hospital, she developed excessive yellowish discharge from her left ear and post auricular swelling along with fever and left ear pain, and was admitted to the hospital under pediatric care. Physical examination revealed an ill-looking patient with a temperature of 39.5 °C, pulse rate of 110 beats/min, respiratory rate of 30/min, and blood pressure of 95/65 mmHg. She was crying and irritable. There was obvious swelling behind her left ear, which was very tender, with yellowish discharge coming out of it. The rest of the physical examination was unremarkable.

**Fig. 1:**
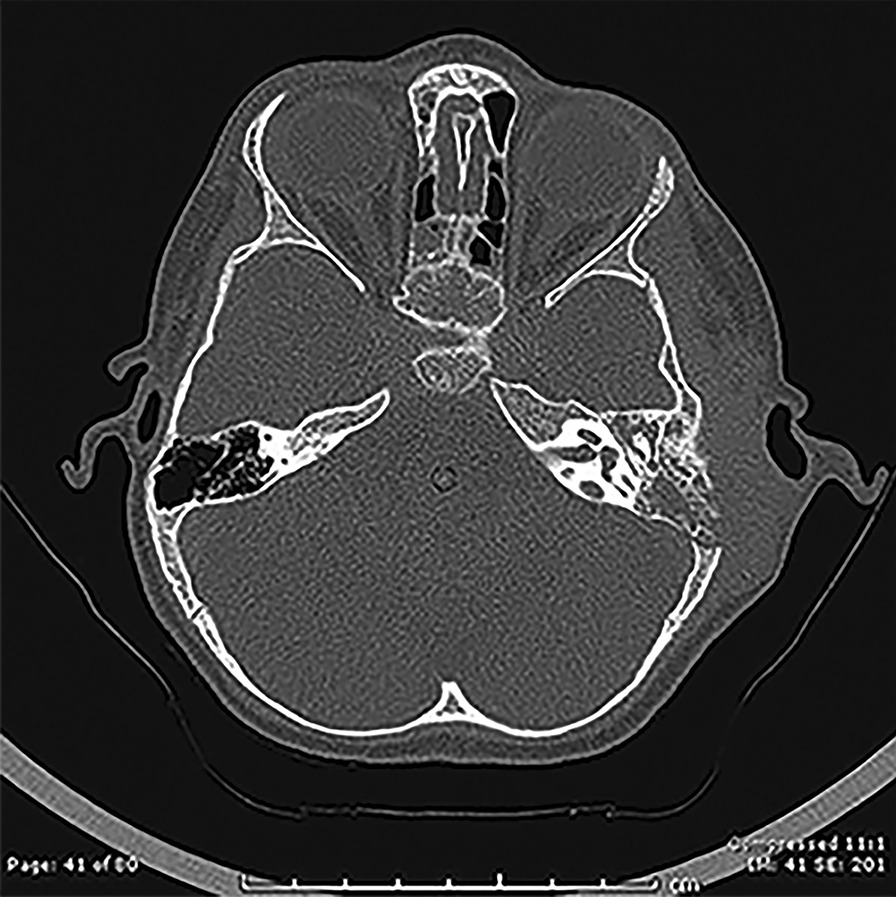
CT scan of the head: showed evidence of left side mastoiditis, opacification of the middle ear cavity and the mastoid air cell, with destruction of the mastoid septae and lateral wall and postauricular swelling

Laboratory workup showed a white blood cell count of 11,400/mm^3^, with lymphocytes 62.1%, eosinophils 4.6%, and neutrophils 25.9%. The inflammatory markers were elevated; erythrocyte sedimentation rate (ESR) of 55 mm/h (reference range 0–13 mm/h) and C-reactive protein (CRP) of 28 mg/L (reference range < 6 mg/L). CT Scan with and without contrast of the head showed evidence of left side mastoiditis, opacification of the middle ear cavity and the mastoid air cell, with destruction of the mastoid septae and lateral wall, and no intracranial extension. In addition, a peripherally filling defect was noted involving the left transverse sinus and was compatible with sinus venous thrombosis secondary to left sided mastoiditis (Fig. 1). Cerebrospinal fluid (CSF) analysis was normal which showed 0 cell WBC, 0 cell RBC, glucose of 3 mmol/L, protein of 20 mg/dL and negative culture. Thrombotic investigations including prothrombine time, partial thromoplastin time, protein C, protein S, lupus anticoagulant factor V leiden by PCR, methylenetetrahydrofolate reductase and prothrombin mutation (factor II) by PCRwere within normal limits. The working diagnosis at that time was acute bacterial mastoiditis complicated with cerebral venous sinus thrombosis, and the patient was empirically managed with intravenous Meropenem and anticoagulant medication (Enoxaparin). The pus sample from her ear revealed significant growth of *Trichosporon asahii* on third day of admission. The sample was tested for identification (ID) and susceptibility by using Vitek 2 Compact. Therefore, Voriconazole 100 mg twice daily intravenously (18 mg/kg/day) was initiated at day three of admission.

During the course of stay in the hospital, the patient became afebrile after 48 h of starting the Voriconzole; post auricular swelling and ear discharge were improved significantly. Despite the aforementioned antibiotic therapy, ear discharge culture remained persistently positive for three occasions and the result was negative at day 7 of admission (4 days after staring Voriconazole). Blood culture did not grow any organism. The Inflammatory markers dropped dramatically (ESR, CRP). Therefore, Meropenem was given for 7 days and Voriconazole intravenously was received for 10 days (6 days after negative ear discharge culture). After 13 days of hospitalization the patient was doing well with no ear discharge. A thorough workup was performed, including flow cytometry (CD markers) and serum immunoglobulins levels. All were unremarkable. She was discharged home on Voriconazole orally for another 3 weeks and was seen at 4 week- outpatient follow up with pediatric infectious diseases care. She was doing very well and her clinical examination and follow up inflammatory markers were normal.

A follow up brain MRI/MRV showed dual venous sinuses which appeared patent with no evidence of venous sinus thrombosis.

## Discussion and conclusion

To the authors’ best knowledge, this is the first case that reports *Trichosporon asahii* mastoiditis which is complicated with cerebral sinus venous thrombosis in an immunocompetent child and with no previous history of hospitalization or any comorbidities. It is well known that the usual causes of acute mastoiditis in immunocompetent children are usually bacteria, includes *Streptococcus pneumoniae*, *Streptococcus pyogenes*, and *Staphylococcus aureus* [7]. In addition, the recent cases of *T. asahii* infection in immunocompetent patients were in adult patients with comorbid diseases and medical intervention [6].

We started Voriconzole by considering the resistance properties of *Trichsporon asahii* and this resulted in swift response and significant improvement in her condition without any remaining complication. Surgical treatments are sometimes performed in combination with medical therapy in cases with very severe complications [7]. The reason why the patient did not need surgery was the significant improvement in her clinical symptoms and signs after starting Voriconazole.

The prolonged multiple antimicrobials used in this patient, may be considered a risk factor for having *T. asahii* infection; as the patient had been prescribed different types of antimicrobial drug and her general condition continued to worsen before admission. Furthermore, there was a delay in seeking appropriate care. *Trichosporon asahii* is non-candida yeast that causes infection in immunosuppressed patients and rarely in immunecompetent children*.* It has been recognized as an opportunistic agent of invasive infections mostly in immunocompromised hosts [8].

*Trichosporon* species are the second most frequently isolated yeast species after Candida species from cancer patients [4]. *T. asahii* can colonize gastrointestinal tract, skin, stool, mucosal surface, central catheter, sputum and hair [1, 4, 6]. The pathophysiology of invasive trichosporonosis involves colonization of the respiratory tract, gastrointestinal tract, or skin, with seeding of the bloodstream through a break in the integrity of a mucosal surface [5].

The infection from this microorganism has a wide spectrum of clinical presentations ranging from cutaneous infection in immunocompetent individual, to disseminated fatal disease in immunocompromised one [1, 2]. Trichosporonosis is usually an insidious disease and its diagnosis can be missed, particularly in developing countries, due to lack of awareness and familiarity with the clinical diagnosis [3].

The diagnosis of *T.asahii* is based on clinical findings and confirmed by microscopy and culture. *T. asahii* is isolated from different clinical specimens. It is usually diagnosed by a specimen obtained from blood, urine and sputum. The most commonly used method for identifying *T. asahii*. is morphological method combined with biochemical methods. These methods include API 20C AUX yeast identification system, ID 32C yeast identification system, and Vitek automatic microbial identification system [9].

Early diagnosis of trichosporonosis remains a challenge and the *Trichosporon species* are less susceptible to some empirical or prophylactic antifungal drugs such as Caspofungin and Amphotericin B [1]. Several studies showed low in vitro sensitivity of *T. asahii* to commonly used antifungal agents [3]. The antifungal activity against *T.asahii* differs between in vivo and in vitro applications; *T. asahii* has reduced susceptibility in vitro to Amphotericin B [1, 2]. The ESCMID/ECMM guidelines in 2014 recommended the use of Voriconazole for the treatment of *T. asahii* infection. The guidelines were based on the results of in vitro susceptibility testing and animal model testing along with supporting evidence from a few case reports of *Trichosporon* infection [9, 10]. Several of the *Trichosporon species* are resistant in vitro to Amphotericin B with MICs ≥ 2 mg/L, including *T. asahii*. *Trichosporon species* are resistant to Flucytosine (MICs 4–128 mg/L) and to the Echinocandins (MICs > 16 mg/L) [11]. Therefore, Voriconazole is the preferred agent as it shows good in vitro activity against most *Trichosporon species* and isolates and also has been associated with good in vivo outcome in most cases of clinical and animal studies [11].

There have been few reported cases of *Trichsporon* infection in immunocompetent adult patient. However, this is the first case of *Trichospsoron* infection causing mastoiditis in immunecompetent child patient. *T. asahii* causing UTI in immunocompetent elderly patients, who were in the ICU and had urine catheter, was reported and successfully treated with Voriconazole [6]. Another case has reported fatal outbreak of *T. asahii* sepsis in eight newborns, in neonatal intensive care unit (NICU) in India.

Acute mastoiditis represents the most common complication of an acute otitis media. Although the disease is rare, it usually affects very young children with severe clinical course and causes serious complications [7]. Streptococcus pneumoniae is considered the predominant pathogen in children who are affected by acute mastoiditis. In the uncomplicated forms of acute mastoiditis, antibiotic therapy is the main treatment. Surgical intervention is performed in combination with medical therapy in cases associated with very severe complications [7]. Therefore, an early and specific antibiotic therapy is fundamental for the resolution of the disease and the prevention of complications.

Despite the recommended treatment of *T. asahii* infection, it is still a fatal one. Despite early treatment with the proper antifungal drugs, mortality rates are still high and could reach up to 80% [4]. Moreover, it is usually not possible to confirm the infection at the early stage. Therefore, early diagnosis and initiating proper empirical treatment properly are very important in reducing the mortality [4, 8].

Optimal diagnosis of *Trichosporon* infection needs a high index of clinical and microbiological suspicion. *T. asahii* can also cause infection in immunocompetent individual even without previous history of hospitalization or intervention. The clinical outcome supports the use of Voriconazole as first line therapy. In addition, early diagnosis and initiating proper therapy are important in minimizing the patient’s mortality.

## Data Availability

All data are contained within the article.

## Abbreviations

*T. asahii*: *Trichosporon asahii*
UTI: Urinary tract infection
MRI: Magnetic Resonance Image
MRV: Magnetic Resonance Venogram
